# The role of *ALDH2* rs671 polymorphism and C-reactive protein in the phenotypes of male ALS patients

**DOI:** 10.3389/fnins.2024.1397991

**Published:** 2024-09-03

**Authors:** Lifang Huang, Mao Liu, Jiahui Tang, Zhenxiang Gong, Zehui Li, Yuan Yang, Min Zhang

**Affiliations:** ^1^Department of Neurology, Tongji Hospital, Tongji Medical College, Huazhong University of Science and Technology, Wuhan, China; ^2^Department of Neurology, SUNY Downstate Health Sciences University, Brooklyn, NY, United States; ^3^Department of Neurology, The First Affiliated Hospital of Xiamen University, Xiamen, China; ^4^Department of Neurology, The Central Hospital of Enshi Tujia and Miao Autonomous Prefecture, Enshi, China; ^5^Department of Neurology, Shanxi Bethune Hospital, Shanxi Academy of Medical Sciences, Tongji Shanxi Hospital, Third Hospital of Shanxi Medical University, Taiyuan, China

**Keywords:** amyotrophic lateral sclerosis, aldehyde dehydrogenase 2, C-reactive protein, disease progression, cognitive impairment

## Abstract

**Background:**

The aldehyde dehydrogenase 2 (*ALDH2*) rs671 (A) allele has been implicated in neurodegeneration, potentially through oxidative and inflammatory pathways. The study aims to investigate the effects of the *ALDH2* rs671 (A) allele and high sensitivity C-reactive protein (hs-CRP) on the clinical phenotypes of amyotrophic lateral sclerosis (ALS) in male and female patients.

**Methods:**

Clinical data and *ALDH2* rs671 genotype of 143 ALS patients, including 85 males and 58 females, were collected from January 2018 to December 2022. All patients underwent assessment using the Chinese version of the Edinburgh Cognitive and Behavioral ALS Screen (ECAS). Complete blood count and metabolic profiles were measured. Clinical and laboratory parameters were compared between carriers and non-carriers of the rs671 (A) allele in males and females, respectively. The significant parameters and rs671 (A) Allele were included in multivariate linear regression models to identify potential contributors to motor and cognitive impairment. Mediation analysis was employed to evaluate any mediation effects.

**Results:**

Male patients carrying rs671 (A) allele exhibited higher levels of hs-CRP than non-carriers (1.70 mg/L vs. 0.50 mg/L, *p* = 0.006). The rs671 (A) allele was identified as an independent risk factor for faster disease progression only in male patients (β = 0.274, 95% CI = 0.048−0.499, *p* = 0.018). The effect of the rs671 (A) allele on the executive function in male patients was fully mediated by hs-CRP (Indirect effect = −1.790, 95% CI = −4.555−−0.225). No effects of the rs671 (A) allele or hs-CRP were observed in female ALS patients. The effects of the *ALDH2* rs671 (A) allele and the mediating role of hs-CRP in male patients remained significant in the sensitivity analyses.

**Conclusion:**

The *ALDH2* rs671 (A) allele contributed to faster disease progression and hs-CRP mediated cognitive impairment in male ALS patients.

## 1 Introduction

Amyotrophic lateral sclerosis (ALS) is a devastating neurodegenerative disease, typically resulting in death within 2 to 5 years from onset (van Es et al., 2017). While this disorder is traditionally characterized by progressive degeneration of upper and lower motor neurons, there is an increasing recognition that ALS exhibits clinical and genetic heterogeneity and forms with frontotemporal dementia (FTD) the so-called Amyotrophic lateral sclerosis–frontotemporal spectrum disorder (ALS-FTSD) (Cividini et al., 2021). Approximately 50% of individuals diagnosed with ALS exhibit varying degrees of cognitive impairment, ranging from mild deficits in cognitive or behavior to severe symptoms characteristic of FTD (Montuschi et al., 2015). The presence of both motor and cognitive abnormalities significantly impacts the quality of life for ALS patients, increases caregiver burden, and reduces patient survival rates (Young et al., 2023).

Over 50 genes have been identified as causative or risk factors for ALS (Bagyinszky et al., 2023). These genetic mutations were related to the motor or cognitive phenotypes of ALS patients, with repeat expansions in the chromosome 9 open reading frame 72 (*C9orf72*) gene-related ALS and FTD serving as a prominent example to date (Bagyinszky et al., 2023). However, unlike the Caucasian population, the prevalence of *C9orf72* mutation remains rather low among the Chinese population (Jiao et al., 2014). However, genetic variations contributing to sporadic ALS, which affects the majority of patients (90–95%), remain largely unknown. Previous studies have demonstrated that the minor allele (C) of *UNC13A* rs12608932 is associated with delayed symptom onset, frequently involving the bulbar function, reduced forced vital capacity at diagnosis, shorter survival time, and an increased risk of frontotemporal dementia in sporadic ALS (Placek et al., 2019; Tan et al., 2020). The minor allele (C) carriers exhibited impaired working memory performance, decreased frontotemporal cortical thickness, and elevated levels of TAR DNA-binding protein-43 (TDP-43) on histopathology (Placek et al., 2019). It is unclear whether other genetic polymorphisms may also influence the motor and cognitive phenotype of ALS patients.

Recent studies have shown the potential role of aldehyde dehydrogenase 2 (*ALDH2*) in Parkinson’s disease (PD) and Alzheimer’s disease (AD). In the Chinese Han population, *ALDH2* genetic variations have been found to increase susceptibility to PD (Zhang et al., 2015). The presence of the *ALDH2* rs671 (A) allele has been associated with an elevated risk of AD (Chen J. et al., 2019), as well as poorer attention and language functions in patients with PD (Yu et al., 2016). The *ALDH2* gene, located at chromosome 12 (12q24) and composed of 13 exons, encodes the ALDH2 protein. This crucial enzyme converts toxic acetaldehyde, a byproduct of ethanol metabolism, into non-toxic acetate. The ALDH2 enzyme activity *in vivo* is significantly decreased by *ALDH2* rs671 (A). While the wild type (GG) exhibits regular activity, heterozygotes (GA) show a decrease in enzyme activity to 10% to 45%, and homozygotes (AA) exhibit a reduction of 1 to 5% (Gross et al., 2015). Additionally, under oxidative stress, ALDH2 plays a role in eliminating endogenous aldehydes such as 4-hydroxy-2-nonenal (4-HNE) and malondialdehyde (MDA), which are implicated in the pathogenesis of neuronal cell death (Yoval-Sanchez and Rodriguez-Zavala, 2012).

Studies on transgenic mouse models have indicated that reducing ALDH2 activity can replicate AD-like and PD-like pathology. Knocking out the *ALDH2* gene or overexpressing the inactive allele has been shown to induce cognitive impairment in mice, with increased 4-HNE deposition, amyloid-β (Aβ) accumulation, tau phosphorylation, and glial cells activation in mouse brain (Ohsawa et al., 2008; D’Souza et al., 2015; Knopp et al., 2020). The double-knockout mouse model for *ALDH2* and aldehyde dehydrogenase 1A1 (*ALDH1A1*) exhibited age-related motor dysfunction accompanied by increased neurotoxic aldehydes including 4-HNE and 3, 4-dihydroxyphenylacetaldehyde (DOPAL), loss of substantia nigra neurons, and decreased dopamine (Wey et al., 2012). The pathological mechanisms involved include exacerbated aldehyde load, oxidative stress, and mitochondrial dysfunction, all playing crucial roles in the occurrence and development of neurodegenerative diseases. 4-HNE impairs glucose and glutamate transport, induces mitochondrial oxidative stress, and leads to mitochondrial dysfunction (Keller et al., 1997). Additionally, 4-HNE enhances Aβ disposition through increased activity of β-secretase and γ-secretase and the formation of an adduct with the amyloid-degrading enzyme neprilysin (Seike et al., 2023). Reactive aldehydes also modify the lysine-rich α-synuclein, resulting in reduced ubiquitination and increased aggregation of neurotoxic oligomers (Plotegher and Bubacco, 2016). Therefore, decreased ALDH2 activity is involved in the pathogenesis of neurodegenerative diseases via various pathways associated with aldehydes accumulation and oxidative stress. Previous *in vitro* studies revealed that oxidative stress was able to promote aggregation of TDP-43 via cysteine oxidation, disulfide bond formation, and acetylation (Cohen et al., 2012, 2015), thereby triggering global mitochondrial imbalance and ultimately causing neuron death in ALS (Zuo et al., 2021). However, it is unclear whether *ALDH2* might play a role in the clinical phenotypes of ALS.

Additionally, previous studies showed that the *ALDH2* rs671 (A) variant was related to metabolic pathways, albeit with some conflicting results. While Imatoh et al. demonstrated a negative correlation between the *ALDH2* rs671 (A) allele and serum high-density lipoprotein cholesterol (HDL-C) level in Japanese male subjects (Imatoh et al., 2018), Hashimoto et al. found no significant correlation between the two (Hashimoto et al., 2002). The rs671 (A) allele was also linked to elevated plasma high-sensitivity C-reactive protein (hs-CRP) levels during the early phase of acute myocardial infarction (Bian et al., 2010), and serum C-reactive protein (CRP) was found as a prognostic biomarker of function and survival in ALS patients (Lunetta et al., 2017). Thus, whether the *ALDH2* rs671 (A) variant might affect the clinical phenotypes of ALS via metabolic and inflammatory pathways also remains unanswered.

Of note, sex-based disparities are observed in the development of ALS. Males were more susceptible than females (Chio et al., 2017), and sex affects the onset and exacerbation of motor and cognitive manifestations of ALS (Chio et al., 2020). Significant sex-related differences in the anatomical patterns of cortical and subcortical pathology were found in ALS patients (Bede et al., 2014). Female SOD1^*G*93*A*^ mice exhibit a delayed disease onset and extended lifespan in comparison to male mice (Pfohl et al., 2015). Sex-dependent outcomes will contribute to the development of tailored patient-specific therapies for ALS. A study on Chinese patients (Chen Y. et al., 2019) found that the *ALDH2* rs671 AA genotype was associated with a 3.99-fold increased risk in ALS compared with the GG genotype in the Chinese Han male population. However, no significant association was found between *ALDH2* gene polymorphism and the risk of ALS in females.

The current study aims to investigate the role of the *ALDH2* rs671 (A) allele in the motor and cognitive function of ALS patients by comparing the clinical characteristics, metabolic profile, and inflammatory marker between *ALDH2* rs671 (A) carriers and non-carriers in a sex-stratified manner.

## 2 Materials and methods

### 2.1 Participants

A total of 143 ALS patients admitted to the Department of Neurology, Tongji Hospital in Wuhan, China, between January 2018 and December 2022 were enrolled in this study. All patients met the diagnostic criteria for possible, probable, or definite ALS per the revised El Escorial criteria (Brooks et al., 2000). Exclusion criteria included: (1) a putative familial background suggestive of ALS (evaluated through comprehensive interviews or genetic testing); (2) the presence of other neurological disorders that could impact motor function or cognition (e.g., cerebrovascular disease, traumatic brain injury, brain tumor, or epilepsy); (3) psychiatric illnesses; (4) history of drug abuse or dependence; (5) coexistence of severe systemic diseases; and (6) incomplete clinical, laboratory or cognitive data. The Ethics Committee of the Tongji Hospital Tongji Medical College of Huazhong University of Science and Technology approved this study (TJ-IRB20201219). Written informed consent was obtained from all participants or their legal representatives before their inclusion.

### 2.2 Clinical data acquisition

Demographic and clinical data, including sex, age, education level, smoking and alcohol history, duration of illness, and site of symptom onset, were collected. The body mass index (BMI) was calculated as weight (kg) divided by squared height (m2). The amyotrophic lateral sclerosis functional rating scale-revised (ALSFRS-R), with a maximum score of 48 points in ALS patients (a lower score suggesting higher disability), was used to assess the degree of physical disability (Cedarbaum et al., 1999). Disease progression rate (PR) was measured using the slope of ALSFRS-R over months since onset [(48−ALSFRS-R score)/months]. Furthermore, the disease stage was evaluated using the King’s staging system based on the involvement of the bulbar region, upper limbs, and lower limbs or the presence of nutritional or respiratory failure: stage 1/2/3 indicates involvement in 1/2/3 body regions, respectively, while stage 4 indicates respiratory or nutritional insufficiency requiring non-invasive ventilation or enteral nutrition (Roche et al., 2012).

### 2.3 Laboratory test

Venous blood samples were collected between 5:00 and 7:00 in the morning following an overnight fast of more than 8 hours. Laboratory parameters, including complete blood count (CBC) and high-sensitivity C-reactive protein (hs-CRP), alanine aminotransferase (ALT), aspartate aminotransferase (AST), total protein (TP), albumin (ALB), urea, creatinine (Cr), uric acid (UA), serum potassium (K^+^), sodium (Na^+^), chlorine ion concentration (Cl^–^), calcium ion concentration (Ca2^+^), total cholesterol (TC), triglyceride (TG), high-density lipoprotein cholesterol (HDL-C), and low-density lipoprotein cholesterol (LDL-C) levels were evaluated one time in the Department of Clinical Laboratory of Tongji Hospital. CBC was performed using XN-9000 Sysmex (Sysmex Co., Kobe, Japan). The biochemical profile including hs-CRP, was conducted on the Cobas 8000 automated system (Roche Diagnostics GmbH, Mannheim, Germany).

### 2.4 Genotyping

Following the manufacturer’s instructions, the DNA was extracted from peripheral blood samples using a whole-blood DNA extraction kit (Sangon, Shanghai, China) and amplified via polymerase chain reaction (PCR). The primer sequences employed were as follows: The forward primer sequence was 5′-CTCGTTTCAAATTACAGGGTCA-3′, and the reverse primer sequence was 5′-TGTCACTTCTCAGGCTTAAAATG-3′. The PCR products were subsequently purified and sent to Sangon Biotech (Shanghai, China) for sequencing using Applied Biosystems 3730XL DNA Analyzer (USA).

### 2.5 Neuropsychological assessment

The Edinburgh Cognitive and Behavioral ALS Screen (ECAS) was designed with high sensitivity and specificity to assess cognitive dysfunction in ALS patients. It consists of a patient-oriented questionnaire that evaluates five domains of cognition and a caregiver-oriented questionnaire that assesses behavioral and psychotic symptoms. The former measures ALS-specific functions (language, verbal fluency, and executive function) and ALS-non-specific functions (memory and visuospatial function), yielding a total score of 136 points. In the Chinese version of ECAS, the cut-off scores for the ECAS cognition total score (81.92 points), ALS-specific score (58.98 points), and ALS-non-specific score (19.70 points) were determined based on two standard deviations below the mean score of the healthy Chinese population (Ye et al., 2016). The caregiver-oriented questionnaire evaluates five behavioral symptoms (disinhibition, apathy, loss of sympathy, perseveration, and changes in eating behaviors) and psychotic symptoms. Behavioral and psychotic data was available for 137 patients due to the absence of caregivers of 6 patients.

### 2.6 Statistical analysis

Normally distributed continuous data were presented as mean ± SD and compared using an independent *t*-test. Non-normally distributed continuous data were described as median (interquartile range) and compared using the Mann-Whitney U test. Categorical variables were reported as frequencies (%) and analyzed using Pearson’s chi-squared or Fisher exact test when appropriate. Demographic and clinical parameters were first included as independent variables in the univariate linear regression models to identify any potential significant contributing factors for ALSFRS-R, PR, and ECAS total and subdomain scores. The *ALDH2* rs671 genotype and significant demographic and laboratory variables were further included in the multivariate linear regression analysis models for male and female patients, respectively. Mediation models evaluating the potential mediating role of laboratory parameters in the relationship between *ALDH2* rs671 polymorphism and motor and cognitive function in ALS were further performed through the SPSS PROCESS macro program (Hayes, 2015) (version 4.0). The analysis employed a bias-corrected bootstrap method with 5,000 bootstrapping resamples to calculate 95% confidence intervals (CI). Statistically significant mediation was determined if the bootstrapping 95% CI did not include zero, and a two-tailed *p*-value of less than 0.05 was considered statistically significant for all other statistical analyses. Two sensitivity analyses were conducted to assess the the robustness of our findings. The first sensitivity analysis was performed to eliminate the potential effect of extreme values, hs-CRP levels outside [Quartile 1 (Q1)−3 x interquartile range (IQR)] or [Quartile 3 (Q) + 3 x IQR] were considered as extreme values and then excluded from the multivariable linear regression models and the mediation analyses. Given that King’s clinical stage 4 is characterized by nutritional or respiratory insufficiency requiring intervention, which could be secondary confounding factors of motor and cognitive phenotypes (Ludolph et al., 2023; Sales de Campos et al., 2023), another sensitivity analysis was restricted to patients in King’s clinical stage 1 to 3. The statistical analysis was conducted using the SPSS Software (version 24.0).

## 3 Results

### 3.1 Demographic and clinical characteristics

**FIGURE 1:**
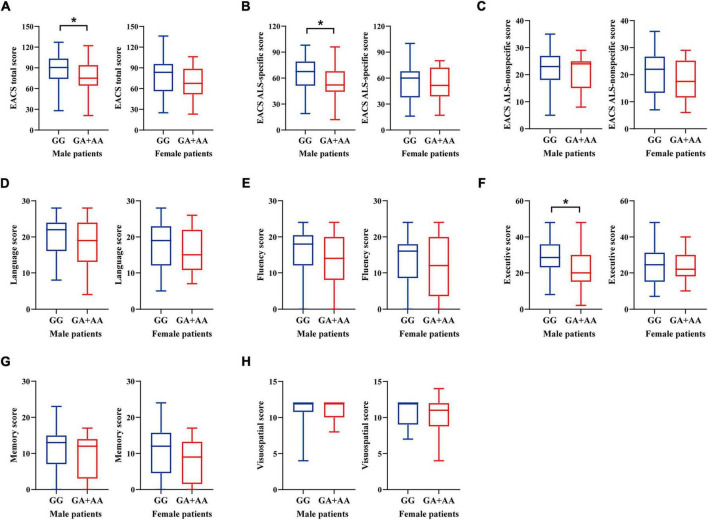
ECAS cognitive scores of ALS patients stratified by sex and *ALDH2* rs671 genotypes. **(A)** ECAS total score. **(B)** ALS-specific score. **(C)** ALS-nonspecific score. **(D)** Language score. **(E)** Fluency score. **(F)** Executive score. **(G)** Memory score. **(H)** Visuospatial score. *p < 0.05.

An overview of the demographic and clinical characteristics of ALS patients stratified by sex and *ALDH2* rs671 genotypes is presented in Table 1. The cohort comprised 85 males (59.44%) and 58 females (40.56%). Among all male patients, 62 had the GG, 22 had the GA, and 1 had the AA genotype. Of all the female patients, 40 had the GG and 18 had the GA genotype while no one had the AA genotype. The genotype distributions did not deviate from Hardy-Weinberg Equilibrium in male (*p* = 1.000) and female patients (*p* = 0.329). Fewer rs671 (A) allele carriers consumed alcohol compared to non-carriers (21.74% vs. 46.77%, *p* = 0.036) in male patients, while no female reported drinking alcohol. Age, education level, smoking status, BMI, site of onset, illness duration, and King’s stages were similar between rs671 (A) allele carriers and non-carriers in either sex group. Additionally, male patients with the rs671 (A) allele exhibited a lower ALSFRS-R score than non-carriers (38 points vs. 42 points, *p* = 0.021). PR was faster among rs671 (A) allele carriers than non-carriers in males, with an average decline rate per month of 1.07 points versus 0.57 points (*p* < 0.001). Among male patients, the rs671 (A) allele carriers performed worse than non-carriers on ECAS with lower total scores (75 points vs. 90.50 points, *p* = 0.026), lower ALS-specific scores (52 points vs. 67.50 points, *p* = 0.016), and lower executive scores (20 points vs. 28.50 points, *p* = 0.010) (Figure 1 and Supplementary Table 1). Moreover, a higher proportion of abnormal ECAS total, ALS-specific, and executive scores were observed among rs671 (A) allele carriers compared to non-carriers in male patients (Supplementary Figure 1). The two groups showed no significant differences in behavioral and psychiatric symptoms (Supplementary Figure 2). Demographic variables, ALSFRS-R score, PR, and ECAS total and subdomain scores did not differ between rs671 (A) allele carriers and non-carriers in female patients (Figure 1, Table 1, Supplementary Table 1, and Supplementary Figures 1, 2).

**TABLE 1:** Demographic and clinical characteristics of ALS patients stratified by sex and *ALDH2* rs671 genotypes.

	Male (*n* = 85)			Female (*n* = 58)		
	GG (*n* = 62)	GA+AA (n = 23)	*P*-value	GG (*n* = 40)	GA+AA (*n* = 18)	*P*-value
Age (year)	53.37 ± 10.97	55.57 ± 8.64	0.390	54.93 ± 10.97	56.67 ± 11.15	0.580
Education (Junior middle School and Below), n (%)	29 (46.77%)	14 (60.87%)	0.248	30 (75%)	13 (72.22%)	1.000
Smoking, n (%)	28 (45.16%)	9 (39.13%)	0.618	1 (2.50%)	0	1.000
Alcohol use, n (%)	29 (46.77%)	5 (21.74%)	**0.036**	0	0	–
BMI	22 ± 2.95	21.99 ± 2.43	0.985	22.53 ± 3.24	22.39 ± 2.11	0.853
Bulbar onset, n (%)	9 (14.52%)	3 (13.04%)	1.000	10 (25%)	5 (27.78%)	1.000
Duration of illness (month)	12 (7.75–19)	8 (6–14)	0.090	9 (7.25–18)	9.50 (4–14)	0.354
ALSFRS-R score	42 (38–44)	38 (33–42)	**0.021**	42 (37–43.75)	39 (37–43.25)	0.484
Progression rate	0.57 (0.26–0.78)	1.07 (0.52–1.67)	**< 0.001**	0.69 (0.26–1.22)	0.96 (0.54–1.43)	0.071
**King’s staging, n (%)**						
1	15 (24.19%)	4 (17.39%)	0.115	12 (30%)	3 (16.67%)	0.253
2	32 (51.61%)	9 (39.13%)		17 (42.50%)	8 (44.44%)	
3	11 (17.74%)	4 (17.39%)		9 (22.50%)	3 (16.67%)	
4	4 (6.45%)	6 (26.09%)		2 (5%)	4 (22.22%)	

ALS, amyotrophic lateral sclerosis; ALDH2, aldehyde dehydrogenase 2; BMI, body mass index; ALSFRS-R, amyotrophic lateral sclerosis functional rating scale-revised. Statistically significant values are highlighted in bold.

### 3.2 The ALDH2 rs671 (A) allele and laboratory parameters

**FIGURE 2:**
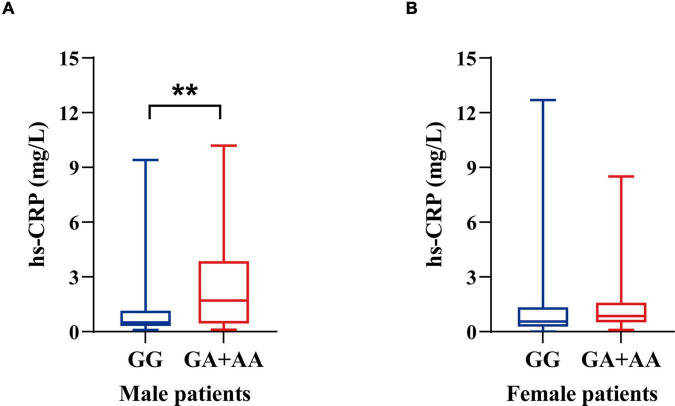
Hs-CRP levels of ALS patients stratified by sex and *ALDH2* rs671 genotypes. **(A)** Hs-CRP levels of male patients. **(B)** Hs-CRP levels of female patients. **p < 0.01.

Compared to non-carriers, male individuals with rs671 (A) allele demonstrated elevated levels of hs-CRP (1.70 mg/L vs. 0.50 mg/L, *p* = 0.006), while no difference was observed between female carriers and non-carriers (0.85 mg/L vs. 0.55 mg/L, *p* = 0.200) (Figure 2 and Supplementary Table 2). No significant differences in other laboratory parameters, including CBC and other parameters of metabolic profile, were observed between the allele carriers and non-carriers in either male or female participants (Supplementary Table 2).

### 3.3 Effects of clinical and laboratory parameters and ALDH2 rs671 (A) allele on the motor and cognitive phenotypes in ALS patients

The duration of illness significantly contributed to the ALSFRS-R score and progression rate in either male or female patients in univariate models (Supplementary Tables 3, 4). For male patients, *ALDH2* rs671 genotypes (β = −0.193, 95% CI = −0.389−0.003, *p* = 0.054) had no independent effect on ALSFRS-R score, while elevated hs-CRP level (β = −0.200, 95% CI = −0.392−−0.008, *p* = 0.041) and longer disease duration (β = −0.528, 95% CI = −0.705−−0.351, *p* < 0.001) were significant predictors of lower ALSFRS-R score (Table 2). The presence of the *ALDH2* rs671 (A) allele (β = 0.274, 95% CI = 0.048−0.499, *p* = 0.018) and shorter disease duration (β = −0.257, 95% CI = −0.459−−0.054, *p* = 0.014) were independently associated with faster progression rate (Table 2). For female patients, both *ALDH2* rs671 genotypes and hs-CRP had no effect on ALSFRS-R or progression rate, while disease duration (β = −0.422, 95% CI = −0.660−−0.184, *p* < 0.001) was negatively associated with progression rate (Supplementary Table 5).

**TABLE 2:** Multivariate linear regression models evaluating the factors influencing the motor and cognitive phenotypes in male patients with ALS.

Independent variables β (95%CI)	ALSFRS-R score	Progression rate	Total score	ALS-specific score	ALS-nonspecific score	Language score	Fluency score	Executive score	Memory score	Visuospatial score
rs671 (A) allele	−0.193 (−0.389–0.003)	**0.274 (0.048**–0.499)*	−0.080 (−0.273–0.113)	−0.103 (−0.289–0.084)	−0.002 (−0.231–0.227)	0.022 (−0.175–0.220)	−0.083 (−0.319–0.154)	−0.133 (−0.327–0.061)	−0.063 (−0.299–0.173)	0.205 (−0.013–0.423)
hs-CRP	−**0.200 (**−**0.392**–−0.008)*	0.117 (−0.103–0.337)	−0.164 (−0.351–0.023)	−**0.183 (**−**0.364**–−0.002)*	−0.074 (−0.297–0.148)	−0.117 (−0.309–0.075)	−0.103 (−0.333–0.127)	−**0.189 (**−**0.378**–−0.0002)*	−0.023 (−0.252–0.206)	−0.209 (−0.421–0.003)
alcohol use	0.078 (−0.104–0.261)	−0.026 (−0.236–0.184)	0.098 (−0.085–0.282)	0.119 (−0.059–0.296)	0.009 (−0.209–0.226)	0.107 (−0.081–0.295)	0.118 (−0.107–0.343)	0.079 (−0.106–0.264)	0.007 (−0.218–0.231)	0.011 (−0.196 – 0.218)
Duration	−**0.528 (**−**0.705**–−0.351)***	−**0.257 (**−**0.459**–−0.054)*	–	–	–	–	–	–	–	–
Age	–	–	−**0.485 (**−**0.659**–−0.311)***	−**0.500 (**−**0.668**–−0.332)***	−**0.321 (**−**0.527**–−0.115)**	−**0.452 (**−**0.631**–−0.274)***	−**0.228 (**−**0.442**–−0.015)*	−**0.483 (**−**0.659**–−0.308)***	−**0.278 (**−**0.491**–−0.065)*	−**0.305 (**−**0.502**–−0.108)**
Education level	–	–	0.263 (0.086 – 0.441)**	0.259 (0.087 – 0.430)**	0.211 (−0.0001 –0.421)	0.350 (0.168 – 0.532)***	0.127 (−0.091–0.345)	0.182 (0.003 – 0.361)*	0.157 (−0.061–0. 374)	0.285 (0.084 – 0.486)**

ALS, amyotrophic lateral sclerosis; ALSFRS-R, amyotrophic lateral sclerosis functional rating scale-revised; hs-CRP: high-sensitivity C-reactive protein.

*p < 0.05,

**p < 0.01,

***p < 0.001. Statistically significant values are highlighted in bold.

The age and education level were significant predictors of ECAS scores in male and female patients, as demonstrated by the univariate models (Supplementary Tables 3, 4). No independent effects of *ALDH2* rs671 allele (A) were observed on ECAS total or subdomain scores in males (Table 2). However, higher hs-CRP significantly predicted lower ALS-specific score (β = −0.183, 95% CI = −0.364−−0.002, *p* = 0.048) and executive score (β = −0.189, 95% CI = −0.378−−0.0002, *p* = 0.0497) while aging and lower educational level independently predicted lower ECAS total and multiple subdomain scores (Table 2). For female patients, *ALDH2* rs671 genotype and hs-CRP did not affect ECAS scores, while aging and lower educational level independently predicted lower ECAS total and multiple subdomain scores (Supplementary Table 5).

### 3.4 The mediating role of C-reactive protein in the effects of ALDH2 rs671 (A) allele on the motor and cognitive function

**FIGURE 3:**
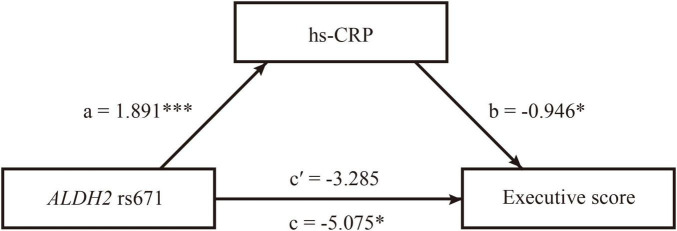
The mediating role of C-reactive protein (CRP) in the relationship between *ALDH2* rs671 (A) allele and executive function in male patients with ALS. The path diagram of the mediation model showed that hs-CRP fully mediated the associations between *ALDH2* rs671 polymorphism and executive function in male patients with ALS. a, the effect of *ALDH2* rs671 (A) allele on hs-CRP; b, the effect of hs-CRP on executive function; c, the total effect of *ALDH2* rs671 (A) allele on executive function; c’, the direct effect of of *ALDH2* rs671 (A) allele on executive function. *p < 0.05, ***p < 0.001.

After adjusting for alcohol consumption and disease duration, the mediation analysis demonstrated that hs-CRP had no mediating effects on the relationship between *ALDH2* rs671 polymorphism and ALSFRS-R and PR in male patients (Table 3). Regarding cognitive function, hs-CRP (Indirect effect = −1.790, 95% CI = −4.555−−0.225) fully mediated the association between *ALDH2* rs671 polymorphism and executive function in male patients after adjusting for alcohol consumption, age, and education level (Table 3 and Figure 3). Hs-CRP was also found to have significant indirect effects on the relationship between *ALDH2* rs671 (A) allele and ECAS total score (Indirect effect = −3.408, 95% CI = −7.637−−0.468) or ALS-specific score (Indirect effect = −3.016, 95% CI = −6.657−−0.602) despite the absence of total effects from *ALDH2* rs671 (A) allele on the respective scores (Table 3). The *ALDH2* rs671 (A) allele exhibited no total effects on ALSFRS-R score, PR, and ECAS total or subdomain scores in female patients (Supplementary Table 6). Neither showed hs-CRP any mediating effects in female patients (Supplementary Table 6).

**TABLE 3:** The mediating role of C-reactive protein in the relationship between *ALDH2* rs671 (A) allele and motor and cognitive function in male patients with ALS.

	ALSFRS-R score	Progression rate	Total score	ALS-specific score	ALS-nonspecific score	Language score	Fluency score	Executive score	Memory score	Visuospatial score
Total effect (95% CI)	−**4.244 (**−**7.073**–−1.415)**	0.498 (0.178 – 0.818)**	−7.743 (−17.560–2.073)	−7.436 (−15.022– 0.150)	−0.439 (−3.495 –2.618)	−0.312 (−2.882 –2.258)	−1.821 (−5.098 –1.456)	−**5.075 (**−**9.615**–−0.535)*	−0.906 (−3.647 –1.835)	0.468 (−0.305 –1.241)
Direct effect (95% CI)	−2.980 (−6.007 –0.047)	0.424 (0.075 – 0.773)*	−4.336 (−14.781–6.110)	−4.420 (−12.443–3.603)	−0.027 (−3.332 –3.278)	0.314 (−2.447 –3.075)	−1.233 (−4.768 –2.303)	−3.285 (−8.089 –1.518)	−0.796 (−3.767 –2.176)	0.769 (−0.050 –1.587)
Indirect effect (95% CI)	−1.264 (−3.117 –0.056)	0.074 (−0.048 –0.229)	−**3.408 (**−**7.637**–−**0.468)**	−**3.016 (**−**6.657**–−**0.602)**	−0.411 (−1.782 –1.061)	−0.626 (−1.666–0.132)	−0.589 (−1.938 –1.052)	−**1.790 (**−**4.555**–−**0.225)**	−0.111 (−1.138 –1.087)	−0.301 (−0.791 –0.111)
Percent mediated (%)	–	–	–	–	–	–	–	100%	–	–

ALDH2, aldehyde dehydrogenase 2; ALS, amyotrophic lateral sclerosis; ALSFRS-R, amyotrophic lateral sclerosis functional rating scale-revised.

* *p* < 0.05,

** *p* < 0.01. Statistically significant values are highlighted in bold.

### 3.5 Sensitivity analyses

After excluding the extreme values of hs-CRP levels, the relationships between *ALDH2* rs671 polymorphism and disease progression, hs-CRP and cognitive function, and the mediating role of hs-CRP in the association between the *ALDH2* rs671 polymorphism and executive function in male patients remained consistent with the primary findings, while the predicting role of hs-CRP in poor motor function was not observed (Tables 4, 5). In another sensitivity analysis restricted to male patients in King’s clinical stage 1 to 3, *ALDH2* rs671 polymorphism was independently associated with disease progression, ALS-specific scores, executive scores, and hs-CRP mediated the relationship between *ALDH2* rs671 polymorphism and cognitive functions to varying degrees. No independent association was found between hs-CRP and motor and cognitive function (Supplementary Tables 7, 8).

**TABLE 4:** Multivariate linear regression models evaluating the factors influencing the motor and cognitive phenotypes in male patients with ALS excluding the extreme values of hs-CRP.

Independent variables β (95%CI)	ALSFRS-R score	Progression rate	Total score	ALS-specific score	ALS-nonspecific score	Language score	Fluency score	Executive score	Memory score	Visuospatial score
rs671 (A) allele	−0.183 (−0.398–0.031)	**0.247 (0.006**–0.487)*	−0.061 (−0.269–0.147)	−0.073 (−0.274–0.129)	−0.020 (−0.266–0.226)	0.034 (−0.182–0.251)	−0.127 (−0.384–0.129)	−0.060 (−0.266–0.146)	−0.082 (−0.337–0.173)	0.214 (−0.017–0.444)
hs−CRP	−0.141 (−0.348–0.067)	0.160 (−0.072–0.393)	−**0.220 (**−**0.418**–−0.022)*	−**0.228 (**−**0.420**–−0.037)*	−0.153 (−0.387–0.081)	−0.162 (−0.368–0.044)	−0.037 (−0.281–0.207)	−**0.280 (**−**0.476**–−0.084)**	−0.084 (−0.327–0.158)	−**0.315 (**−**0.535**–−0.096)**
alcohol use	0.048 (−0.145–0.241)	−0.012 (−0.228–0.204)	0.104 (−0.086–0.295)	0.140 (−0.044–0.323)	−0.033 (−0.258–0.192)	0.118 (−0.080–0.316)	0.094 (−0.140–0.329)	0.124 (−0.064–0.312)	−0.023 (−0.256–0.210)	−0.050 (−0.261–0.161)
Duration	−**0.544 (**−**0.730**–−0.358)***	−**0.262 (**−**0.471**–−0.054)*	–	–	–	–	–	–	–	–
Age	–	–	−**0.483 (**−**0.661**–−0.305)***	−**0.507 (**−**0.679**–−0.335)***	−**0.298 (**−**0.508**–−0.088)**	−**0.447 (**−**0.632**–−0.262)***	−**0.234 (**−**0.453**–−0.015)*	−**0.495 (**−**0.671**–−0.318) ***	−**0.257 (**−**0.474**–−0.039)*	−**0.280 (**−**0.477**–−0.083)**
Education level	–	–	0.260 (0.078 – 0.442)**	0.258 (0.082 – 0.434)**	0.202 (−0.013 – 0.417)	0.341 (0.152 – 0.531)***	0.118 (−0.106 – 0.342)	0.192 (0.012 – 0.373)*	0.144 (−0.079 – 0.367)	0.298 (0.096 – 0.500)**

ALS, amyotrophic lateral sclerosis; ALSFRS-R, amyotrophic lateral sclerosis functional rating scale-revised; hs-CRP: high-sensitivity C-reactive protein.

* *p* < 0.05,

** *p* < 0.01,

*** *p* < 0.001. Statistically significant values are highlighted in bold.

**TABLE 5:** The mediation analyses of hs-CRP in the relationship between *ALDH2* rs671 (A) allele and motor and cognitive function in male patients with ALS excluding the extreme values of hs-CRP.

	ALSFRS-R score	Progression rate	Total score	ALS-specific score	ALS-nonspecific score	Language score	Fluency score	Executive score	Memory score	Visuospatial score
Total effect (95% CI)	−**3.844 (**−**6.761**–−0.927)*	**0.516 (0.176**–0.856)**	−9.106 (−19.600–1.388)	−7.925 (−16.036–0.185)	−1.330 (−4.505–1.844)	−0.601 (−3.349 –2.147)	−2.203 (−5.664 –1.259)	−**4.864 (**−**9.727–**−0.002)*	−1.559 (−4.456 –1.339)	0.228 (−0.538 –0.995)
Direct effect (95% CI)	−2.804 (−6.088 –0.480)	0.393 (0.010 –0.776)*	−3.367 (−14.836 –8.102)	−3.180 (−12.002–5.643)	−0.286 (−3.827 –3.255)	−0.481 (−2.569 –3.531)	−1.934 (−5.837 –1.968)	−1.509 (−6.719–3.702)	−1.047 (−4.304–2.211)	−0.760 (−0.061 –1.581)
Indirect effect (95% CI)	−1.040 (−2.900 –0.406)	0.123 (−0.063 –0.335)	−**5.739 (**−**12.441**–−**0.921)**	−**4.745 (**−**9.860**–−**0.982)**	−1.044 (−2.982–0.381)	−**1.082 (**−**2.529–**−**0.010)**	−0.268 (−2.826 –1.781)	−**3.356 (**−**6.492**–−**0.784)**	−0.512 (−2.105 –0.809)	−**0.532 (**−**1.017**–−**0.139)**
Percent mediated (%)	–	–	–	–	–	–	–	100%	–	–

hs-CRP: high-sensitivity C-reactive protein; ALDH2, aldehyde dehydrogenase 2; ALS, amyotrophic lateral sclerosis; ALSFRS-R, amyotrophic lateral sclerosis functional rating scale-revised.

* *p* < 0.05,

** *p* < 0.01. Statistically significant values are highlighted in bold.

## 4 Discussion

For the first time we showed that *ALDH2* rs671 (A) allele male carriers had poorer motor and cognitive function along with a more rapid progression rate. *ALDH2* rs671 (A) allele served as an independent risk factor for faster progression, while hs-CRP independently predicted worsening motor function and ALS-specific cognitive function, including executive function, in male patients with ALS. In addition, hs-CRP fully mediated the effects of the *ALDH2* rs671 (A) allele on executive function. In contrast, neither *ALDH2* rs671 (A) nor hs-CRP showed any predicting effects on motor or cognitive function in female patients with ALS. Additionally, the effects of disease duration on motor phenotypes and age and educational level on cognitive function were observed in both male and female patients. The effect of the *ALDH2* rs671 (A) allele in progression rate, and the mediating role of hs-CRP in the relationship between the *ALDH2* rs671 (A) allele and cognitive function in male patients, remained statistically significant in the sensitivity analyses. However, further validation is need to confirm the predictive role of hs-CRP in poor motor and cognitive function. It is suggested that the effects of hs-CRP on phenotypes in male patients may be limited to inflammation conditions to some extent and influenced by disease stages.

The *ALDH2* rs671 (A) allele is more prevalent in East Asians than Caucasians (Goedde et al., 1992). This allele is associated with reduced enzyme activity, accumulating acetaldehyde during alcohol metabolism, and manifesting as the well-known alcohol flush reaction characterized by facial flushing, nausea, and rapid heartbeat (Takeshita et al., 2001). Consequently, this genetic variant reduces the likelihood of heavy drinking and alcohol dependence (Luczak et al., 2006), which aligns with our finding that male individuals carrying the rs671 (A) allele were less inclined to consume alcohol.

Previous studies have yielded diverse results when investigating the impact of *ALDH2* polymorphisms on neurodegenerative diseases. For instance, Kamino et al. reported the association between *ALDH2* rs671 polymorphism and late-onset AD (LOAD) in a Japanese cohort with an average age of 76.7 years (Kamino et al., 2000). Similarly, a study on 1949 Chinese individuals aged 90 years and older found that *ALDH2* rs671 polymorphism was associated with cognitive dysfunction independent of alcohol consumption (Jin et al., 2021). However, conflicting findings were observed in other studies. Specifically, one cross-sectional study of 690 Koreans (Kim et al., 2004) and another observational study of 510 Koreans (Shin et al., 2005) did not find any association between *ALDH2* rs671 polymorphism and AD in individuals aged 65 years and older. Furthermore, a Japanese study indicated that the *ALDH2* rs671 polymorphism did not modify the risk of AD (Komatsu et al., 2014). In addition to its impact on AD, certain *ALDH2* polymorphisms have also been implicated in PD. For instance, Zhang et al. and Zhao et al. found that the *ALDH2* rs671 and rs4767944 polymorphisms were associated with an increased risk of PD in the Chinese Han population (Zhang et al., 2015; Zhao et al., 2016); however, the association between *ALDH2* rs4767944 polymorphism and PD was not observed in Iranian (Madadi et al., 2016). Moreover, alcohol consumption is generally considered a modifier of neurodegenerative diseases, although results are not entirely consistent (Peng et al., 2020). Alcohol exposure with reduced ALDH2 activity and metabolism of toxic substances could be involved in the pathogenesis of neurodegenerative diseases. The discrepancies among previous studies’ findings may be attributed to differences in study design, population age, genetic variabilities across ethnic groups, and cultural and societal contexts.

Genetic factors have been demonstrated to influence ALS progression. The *C9orf72* mutation has been linked to a faster progression rate (Mandrioli et al., 2023), while certain *SOD1* mutations have been associated with a slower progression rate (Tang et al., 2021). In addition to disease-causing genes, some gene polymorphisms may act as disease modifiers in ALS, affecting the onset, progression rate, and survival of the disease. For instance, the *UNC13A* rs12608932 polymorphism has been linked to an increased risk for both ALS and FTD (Vidal-Taboada et al., 2015; Tan et al., 2020) and interleukin 1 beta (*IL-1β*) rs1071676 polymorphism is involved in inflammation (Ravnik-Glavac et al., 2022). In the present study, we found that the *ALDH2* rs671 polymorphism was associated with a more rapid progression rate in male patients with ALS. Studies have revealed that toxic aldehyde accumulation is a common pathological feature in neurodegenerative diseases (Li et al., 2022). The rs671 (A) allele decreases ALDH2 enzyme activity and impairs the metabolism of toxic acetaldehyde, which could potentially aggravate oxidative stress injury and eventually result in accelerated neuronal death. Moreover, Alda-1, an ALDH2 agonist, has been found to promote microglia polarization from an inflammatory M1 to an anti-inflammatory M2 status, both *in vivo* and *in vitro* (Zhang et al., 2020). Ada-1 also attenuated sepsis-induced brain injury by regulating NOD-, LRR-, and pyrin domain-containing protein 3 (NLRP3) inflammasome activation (Ling et al., 2023). Silencing the *ALDH2* gene exacerbated vascular inflammation and instability of atherosclerotic plaques in ApoE^–/–^ mice (Pan et al., 2016). While significantly fewer male rs671 (A) allele carriers consumed alcohol than their non-carrier counterparts and we could not measure the actual amount of average alcohol use, the associated aldehyde-induced oxidative damage remains only as a presumption; however, rs671 (A) allele mediated inflammatory response could be a possible explanation for faster disease progression.

CRP, a well-established and highly sensitive systemic biomarker of inflammation, is synthesized in hepatocytes in response to various inflammatory cytokines, including interleukin 6 (IL-6), interleukin 1(IL-1) and tumor necrosis factor-alpha (TNF-α) (Du Clos, 2000). Hs-CRP is an even more sensitive measurement of CRP, enabling quantification of the protein even at deficient levels. Our findings indicate that male carriers of rs671 (A) allele exhibit higher hs-CRP concentrations than non-carriers. The *ALDH2* rs671 (A) allele was associated with high levels of hs-CRP in both acute coronary syndrome patients and control subjects (Bian et al., 2010; Xu et al., 2011). As alluded before, the presence of the rs671 (A) allele might lead to diminished enzymatic activity, further contributing to enhanced inflammatory response. Therefore, increased CRP level could be a downstream signal of the inflammatory pathway. Inflammation plays a crucial role in the pathogenesis of ALS. Several inflammatory markers have been reported to be associated with the progression rate of ALS. These include IL-5, IL-13, IL-18 (Xu et al., 2024), soluble cluster differentiation 14, lipopolysaccharide binding protein (Beers et al., 2020), IL-1β (Jin et al., 2020), neutrophil to lymphocyte ratio (Wei et al., 2022), and the neuroinflammation biomarker osteopontin (Ju et al., 2024). The majority of studies investigating CRP levels in ALS have demonstrated higher CRP levels in ALS patients compared to healthy controls (Kharel et al., 2022). Elevated blood CRP was associated with increased disease severity, faster progression rate, and shorter survival in ALS patients (Lunetta et al., 2017). In a Phase 2 clinical trial for NP001, a monocyte and macrophage regulator, CRP levels were identified as a pharmacodynamic marker for ALS treatment response (Lunetta et al., 2017).

Of note, our findings also indicate that hs-CRP fully mediates the effects of rs671 (A) allele on executive function in male ALS patients. While previous studies have reported associations between serum CRP level and cognitive impairment under various conditions (Long et al., 2023; Wang et al., 2023), our research is the first one showing the adverse effects of serum hs-CRP level on the cognitive function of ALS patients. Given its role as an activator of the complement system, it is postulated that CRP may activate the classical complement pathway and contribute to the development of ALS (Wolbink et al., 1996; Goldknopf et al., 2006). Furthermore, being a systemic inflammatory marker, CRP could potentially enhance the permeability of the blood-brain barrier (BBB) and facilitate the transport of inflammatory cytokines across BBB (Hsuchou et al., 2012). Consequently, activated microglia could promote cytotoxicity by secreting pro-inflammatory cytokines, including IL-1, IL-6, and TNF-α (Beers and Appel, 2019), and lead to neuronal death. Thus, the mediating role of CRP in the effects of the *ALDH2* rs671 (A) allele on executive function in male ALS patients could be plausibly explained by its function in the inflammatory cascade.

Surprisingly, the effects of rs671 (A) allele and hs-CRP on motor and cognitive function were only observed in male patients. A meta-analysis of five studies conducted in Asia found that the *ALDH2* rs671 (A) allele increased the risk of LOAD only in males (Liu et al., 2020). One study on patients with ST-elevation myocardial infarction indicated that the *ALDH2* rs671 (A) allele was associated with more severe myocardial ischemia/reperfusion injury only in male patients (Ishida et al., 2022). A Chinese study found that the *ALDH2* rs10744777 (CT/TT) genotypes were independent risk factors for ischemic stroke in males, and the *ALDH2* rs886205 GA genotype was related to poorer prognosis exclusively in male patients with ischemic stroke (Cheng et al., 2018). Consistently, a recent multi-ancestry meta-analysis reported the association between the *ALDH2* locus and ischemic stroke with significant sex heterogeneity (Surakka et al., 2023). The association was male-specific and observed only in those with East Asian ancestry. However, the sex-specific mechanisms involving *ALDH2* polymorphisms remain unclear. Some researchers have suggested variations in alcohol consumption patterns between males and females in specific cultural and societal contexts may play a role in these differences (Surakka et al., 2023). A previous animal study has demonstrated that female hearts exhibit increased phosphorylation and activity of ALDH2, leading to reduced oxidative stress and, consequently, less ischemia and reperfusion injury compared to male hearts (Lagranha et al., 2010). It has been suggested that the increase in ALDH2 phosphorylation may be mediated by the estrogen-activated phosphatidylinositol 3-kinase (PI3K) pathway (Lagranha et al., 2010). Substantial evidence also indicates that females are more resistant to the effects of reactive oxygen species (ROS) than males through various mechanisms, including female hormones influencing redox homeostasis, potential effects of sex chromosome composition on gene expression related to ROS responses, and the increased optimization of the functional synergy between mitochondrial genes and nuclear genomic asset in females (Tiberi et al., 2023). Therefore, it is plausible that males may be more susceptible to aldehyde-induced oxidative damage than females in ALS. Additionally, previous studies have reported sex differences in inflammation response and regulation in ALS (Trojsi et al., 2020; Grassano et al., 2023). Sex hormones likely play a role in these observed differences: estrogens are known to have anti-inflammatory effects (Straub, 2007), which can inhibit IL-6 production and gene expression (Liu et al., 2005), thereby reducing CRP levels. Treatment with 17β-Estradiol in male SOD1^G93A^ mice increased motoneuron survival by downregulating NLRP3 inflammasome components, including IL1β expression (Heitzer et al., 2017), suggesting protective effects of female hormone against inflammation.

There were several limitations to the present study. Firstly, it is a single-center cross-sectional study, which may introduce potential inclusion bias. Secondly, a larger sample size in male patients might have increased the statistical power to detect differences in motor and cognitive performance between rs671 (A) allele carriers and non-carriers. Thirdly, we did not include more inflammatory markers to better elucidate detailed changes in the inflammatory pathways. Furthermore, we did not screen for genetic mutations including *C9orf72* repeat expansion in our cohort, which could exhibit unique ALS phenotypes; however, the frequency of *C9orf72* repeat expansion in Chinese ALS patients is extremely low. Meanwhile, the frequency of rs671 (A) allele carrier status is much lower outside East Asia, limiting the generalizability of our findings. Additionally, lifestyle behaviors and cardiovascular risk factors could contribute to neurodegenerative diseases including ALS and AD (Huang et al., 2023); our study did not explore the details including the frequency and quantity of substance use and exercises, and other comorbidities such as hypertension and diabetes were not comprehensively evaluated. Lastly, repeated measures of hs-CRP levels could have increased the data accuracy and consistency, taking into account the natural variability of hs-CRP levels over time. Therefore, large-scale, international, multicenter and longitudinal studies are needed to confirm our findings in the future.

## 5 Conclusions

The *ALDH2* rs671 (A) allele was identified as an independent risk factor for accelerated disease progression, and the association between the *ALDH2* rs671 (A) allele and cognitive function was mediated by hs-CRP only in male ALS patients. Further investigations are warranted to elucidate the precise contribution of this allele to ALS pathology in males, potentially paving the road to novel therapeutic strategies that could target inflammation and sexual differences.

## Data Availability

The data presented in the study are deposited in the SRA repository, accession number PRJNA1147393.
