# Supplementary material for: The role of ALDH2 rs671 polymorphism and C-reactive protein in the phenotypes of male ALS patients
Source: Front Neurosci. 2024 Sep 3;18:1397991. doi: 10.3389/fnins.2024.1397991 (PMC11405379; doi:10.3389/fnins.2024.1397991)
Supplement: Supplementary file 1 [file Table_1.docx]

Supplementary Material

**Supplementary Figure 1** Comparison of the frequency of abnormal ECAS scores between ALS patients carrying the *ALDH2* rs671 (A) allele and non-carriers in males **(A)** and females **(B)**

**Supplementary Figure 2** Comparison of the frequency of impaired ECAS behavioral domains between ALS patients carrying the *ALDH2* rs671 (A) allele and non-carriers in males **(A)** and females **(B)**

**Supplementary Table 1** Comparison of ECAS cognitive scores between ALS patients carrying the *ALDH2* rs671 (A) allele and non-carriers

|  | **Male(n=85)** | | | **Female(n=58)** | | |
| --- | --- | --- | --- | --- | --- | --- |
|  | **GG (n=62)** | **GA+AA (n=23)** | ***P*-value** | **GG (n=40)** | **GA+AA (n=18)** | ***P*-value** |
| **Total score** | 90.50 (73.75-103.50) | 75 (64-94) | **0.026** | 83.50 (56-95.75) | 67.50 (51.50-88.75) | 0.373 |
| **ALS-specific score** | 67.50 (51-79.25) | 52 (44-68) | **0.016** | 60 (37.50-68) | 51.50 (38.75-72.25) | 0.620 |
| **Language score** | 22 (16-24) | 19 (13-24) | 0.319 | 19 (12-23) | 15 (10.75-22) | 0.372 |
| **Fluency score** | 18 (12-20.50) | 14 (8-20) | 0.143 | 16 (8.50-18) | 12 (3.50-20) | 0.722 |
| **Executive score** | 28.50 (23-36) | 20 (15-30) | **0.010** | 24.50 (15-31.25) | 22 (18-30) | 0.788 |
| **ALS-nonspecific score** | 23 (18-27) | 24 (15-25) | 0.475 | 22 (13.25-26.75) | 17.50 (11.50-25.25) | 0.225 |
| **Memory score** | 13 (7-15) | 12 (3-14) | 0.218 | 12 (4.50-15.75) | 9 (1.50-13.25) | 0.214 |
| **Visuospatial score** | 12 (10.75-12) | 12 (10-12) | 0.910 | 12 (9-12) | 11 (8.75-12) | 0.679 |

ECAS, Edinburgh Cognitive and Behavioral ALS Screen; ALS, amyotrophic lateral sclerosis; ALDH2, aldehyde dehydrogenase 2.

**Supplementary Table 2** Laboratory parameters of ALS patients stratified by sex and *ALDH2* rs671 genotypes

|  | **Male** | | | **Female** | | |
| --- | --- | --- | --- | --- | --- | --- |
|  | **GG** | **GA+AA** | ***P*** | **GG** | **GA+AA** | ***P*** |
| **WBC (×10^9^/L)** | 5.46 (4.75-6.12) | 5.56 (4.16-6.24) | 0.909 | 4.95 (4.44-5.89) | 4.69 (3.99-6.14) | 0.534 |
| **RBC (×10^12^/L)** | 4.52 (4.19-4.86) | 4.48 (4.29-4.83) | 0.933 | 4.14 (3.91-4.33) | 4.07 (3.77-4.32) | 0.321 |
| **Platelet (×10^9^/L)** | 189.50  (160.50-232.25) | 192  (164-234) | 0.894 | 215  (176.50-268) | 233  (200-284.75) | 0.271 |
| **hs-CRP (mg/L)** | 0.50 (0.27-1.20) | 1.70 (0.40-3.90) | **0.006** | 0.55 (0.23-1.38) | 0.85 (0.48-1.63) | 0.200 |
| **ALT (U/L)** | 21.50 (15-29) | 21 (17-29) | 0.733 | 14 (12-26) | 17.50 (12-22.75) | 0.438 |
| **AST (U/L)** | 21 (17-25.25) | 21 (19-26) | 0.475 | 17 (15-22) | 20 (17-27) | 0.067 |
| **TP (g/L)** | 66.15  (64.68-68.68) | 65.90  (62.80-69.20) | 0.576 | 66.85  (62.55-69.70) | 68.60  (64.30-70.75) | 0.243 |
| **ALB (g/L)** | 41.25  (38.60-42.73) | 39.60  (36.90-42.20) | 0.064 | 40.10  (38.93-41.50) | 40.80  (39.28-42.33) | 0.329 |
| **Urea (mmol/L)** | 5.60 (4.81-6.38) | 5.50 (4.87-6.24) | 0.984 | 5.23 (4.15-6.64) | 4.44 (3.78-5.95) | 0.052 |
| **Cr (umol/L)** | 65 (58-73.25) | 65 (56-72) | 0.403 | 51.50 (46-62) | 51.50 (46-58.75) | 0.656 |
| **UA (umol/L)** | 336.50  (292.75-389.25) | 321  (265-376) | 0.168 | 247.50  (218.75-306) | 250  (208.25-310) | 0.847 |
| **K^+^ (mmol/L)** | 3.93 (3.68-4.14) | 3.96 (3.78-4.02) | 0.925 | 3.89 (3.63-4.13) | 3.86 (3.63-4.06) | 0.730 |
| **Na^+^ (mmol/L)** | 141.70  (140.90-143) | 141.40  (140.40-142.80) | 0.566 | 142.10  (140.63-142.78) | 141.50  (140.90-142.98) | 0.893 |
| **Cl^-^ (mmol/L)** | 103.15 (102.48-104.43) | 104 (102.60-105.70) | 0.411 | 104.70 (103.52-105.77) | 103.80 (101.18-105.20) | 0.210 |
| **Ca^2+^ (mmol/L)** | 2.28 (2.24-2.30) | 2.26 (2.21-2.34) | 0.670 | 2.26 (2.19-2.33) | 2.29 (2.24-2.39) | 0.145 |
| **TC (mmol/L)** | 3.75 (3.41-4.29) | 4.19 (3.86-4.45) | 0.068 | 4.28 (3.65-4.99) | 4.35 (3.72-5.12) | 0.712 |
| **TG (mmol/L)** | 1.16 (0.86-1.66) | 1.13 (0.94-1.94) | 0.656 | 1.26 (0.88-1.94) | 1.49 (1.08-1.94) | 0.342 |
| **HDL-C(mmol/L)** | 1.04 (0.93-1.26) | 0.93 (0.82-1.32) | 0.455 | 1.17 (0.99-1.25) | 1.27 (0.93-1.47) | 0.342 |
| **LDL-C (mmol/L)** | 2.40 (1.97-2.93) | 2.60 (2.34-3.11) | 0.124 | 2.75 (2.27-3.45) | 2.54 (1.92-3.19) | 0.480 |

ALS, amyotrophic lateral sclerosis; ALDH2, aldehyde dehydrogenase 2; WBC, white blood cell; RBC, red blood cell; hs-CRP, high-sensitivity C-reactive protein; ALT, alanine aminotransferase; AST, aspartate aminotransferase; TP, total protein; ALB, albumin; Cr, creatinine; UA, uric acid; K^+^, potassium; Na^+^, sodium; Cl^-^, chlorine; Ca^2+^, calcium; TC, total cholesterol; TG, triglyceride; HDL-C, high-density lipoprotein cholesterol; LDL-C, low-density lipoprotein cholesterol.

**Supplementary Table** **3** Univariate linear regression analyses identifying potential factors influencing motor and cognitive functions in male patients with ALS

| **Independent variables**  **β(95%CI)** | **ALSFRS-R**  **score** | **Progression**  **rate** | **Total score** | **ALS-specific score** | **ALS-nonspeci-fic score** | **Language score** | **Fluency score** | **Executive score** | **Memory score** | **Visuosp-atial score** |
| --- | --- | --- | --- | --- | --- | --- | --- | --- | --- | --- |
| **Age** | -0.190  (-0.405 – 0.024) | 0.206  (-0.008 – 0.419) | **-0.541**  **(-0.725 – -0.358)***** | **-0.559**  **(-0.740 – -0.378)***** | **-0.358**  **(-0.562 – -0.154)***** | **-0.504**  **(-0.693 –**  **-0.316) ***** | **-0.258**  **(-0.469 – -0.047)*** | **-0.538**  **(-0.722 – -0.354)***** | **-0.307**  **(-0.515 – -0.099)**** | **-0.349**  **(-0.554 – -0.145)**** |
| **Education level** | -0.088  (-0.306 – 0.129) | -0.036  (-0.254 – 0.182) | **0.341**  **(0.135 – 0.546)**** | **0.340**  **(0.135– 0.545)**** | **0.261**  **(0.051– 0.472)*** | **0.403**  **(0.203 – 0.603)***** | 0.159  (-0.056 – 0.375) | **0.273**  **(0.063– 0.483)*** | 0.203  (-0.010 – 0.417) | **0.325**  **(0.118– 0.531)**** |
| **Smoking** | -0.130  (-0.346 – 0.087) | -0.136  (-0.081 –  0.352) | -0.044  (-0.262 – 0.174) | -0.016  (-0.234 – 0.202) | -0.113  (-0.330 – 0.104) | -0.073  (-0.290 – 0.145) | 0.054  (-0.164 – 0.272) | -0.023  (-0.241 – 0.195) | -0.096  (-0.313 – 0.121) | -0.114  (-0.331 – 0.103) |
| **Alcohol use** | 0.081  (-0.137 –  0.298) | -0.087  (-0.304 – 0.131) | 0.018  (-0.200 – 0.236) | 0.041  (-0.177 – 0.259) | -0.059  (-0.277 – 0.159) | -0.005  (-0.224 –  0.213) | 0.087  (-0.131 – 0.304) | 0.023  (-0.195 –  0.241) | -0.029  (-0.247 – 0.189) | -0.130  (-0.346 – 0.087) |
| **BMI** | 0.200  (-0.013 –  0.414) | 0.026  (-0.192 – 0.245) | 0.018  (-0.200 –  0.236) | 0.021  (-0.198 –  0.239) | 0.007  (-0.211 – 0.225) | -0.065  (-0.283 –  0.153) | 0.009  (-0.209 – 0.227) | 0.068  (-0.150 –  0.286) | 0.003  (-0.215 – 0.222) | 0.016  (-0.202 – 0.234) |
| **Bulbar onset** | 0.160  (-0.056 –  0.375) | -0.161  (-0.376 – 0.055) | 0.025  (-0.194 –  0.243) | 0.001  (-0.220 –  0.217) | 0.098  (-0.119 – 0.316) | 0.133  (-0.083 –  0.350) | -0.162  (-0.377 – 0.054) | 0.018  (-0.200 –  0.236) | 0.077  (-0.141 – 0.294) | 0.122  (-0.095 –  0.338) |
| **Duration of illness** | **-0.521**  **(-0.708 –**  **-0.335)***** | **-0.276**  **(-0.486 –**  **-0.066)*** | 0.058  (-0.160 – 0.275) | 0.028  (-0.190 – 0.246) | 0.135  (-0.081 – 0.352) | 0.035  (-0.183 –  0.253) | 0.083  (-0.135 – 0.300) | -0.023  (-0.241 –  0.195) | 0.144  (-0.073 – 0.360) | 0.040  (-0.178 –  0.258) |

ALS, amyotrophic lateral sclerosis; CI, confidence interval; BMI, body mass index; ALSFRS-R, amyotrophic lateral sclerosis functional rating scale-revised. * *p* < 0.05, ** *p* < 0.01, *** *p* < 0.001.

**Supplementary Table 4** Univariate linear regression analyses identifying potential factors influencing motor and cognitive functions in female patients with ALS

| **Independent**  **variables**  **β(95%CI)** | **ALSFRS-R**  **score** | **Progression**  **rate** | **Total score** | **ALS-specific score** | **ALS-nonspeci-fic score** | **Language score** | **Fluency score** | **Executive score** | **Memory score** | **Visuosp-atial score** |
| --- | --- | --- | --- | --- | --- | --- | --- | --- | --- | --- |
| **Age** | -0.093  (-0.360 –  0.174) | -0.141  (-0.406 –  0.124) | -0.168  (-0.432 –  0.096) | -0.155  (-0.420 – 0.109) | -0.185  (-0.448 – 0.078) | **-0.289**  **(-0.546 –**  **-0.033)*** | 0.020  (-0.248 – 0.287) | -0.137  (-0.402 –  0.128) | -0.240  (-0.500 – 0.020) | -0.091  (-0.175 – 0.358) |
| **Education level** | 0.121  (-0. 145–  0.386) | -0.146  (-0.411 –  0.119) | **0.443**  **(0.203 –**  **0.683)***** | **0.419**  **(0.176 – 0.662)**** | **0.396**  **(0.151 –**  **0.642)**** | **0.383**  **(0.136 –**  **0.630)**** | **0.339**  **(0.087 – 0.590)**** | **0.406**  **(0.161 –**  **0.650)**** | **0.345**  **(0.094 – 0.596)**** | **0.124**  **(0.119 –**  **0.617)**** |
| **Smoking** | 0.139  (-0.126 –  0.404) | -0.137  (-0.402 –  0.128) | 0.085  (-0.182 –  0.352) | 0.075  (-0.192 – 0.342) | 0.100  (-0.166 – 0.366) | 0.098  (-0.168 –  0.365) | 0.017  (-0.251 – 0.284) | 0.077  (-0.190 –  0.344) | 0.105  (-0.161 – 0.371) | 0.038  (-0.230 – 0.305) |
| **BMI** | 0.028  (-0.239 –  0.296) | -0.106  (-0.372 –  0.160) | -0.085  (-0.352 –  0.181) | -0.075  (-0.342 – 0.191) | -0.074  (-0.341 – 0.193) | -0.121  (-0.387 –  0.145) | -0.077  (-0.344 – 0.189) | -0.037  (-0.304 –  0.231) | -0.162  (-0.427 – 0.102) | 0.253  (-0.006 – 0.512) |
| **Bulbar onset** | -0.033  (-0.300 –  0.235) | -0.048  (-0.315 –  0.219) | 0.052  (-0.215 –  0.320) | 0.054  (-0.214 – 0.321) | 0.004  (-0.264 – 0.271) | 0.074  (-0.193 –  0.341) | 0.074  (-0.193 – 0.341) | 0.028  (-0.240 –  0.296) | -0.024  (-0.292 – 0.244) | 0.114  (-0.152 – 0.380) |
| **Duration of illness** | -0.161  (-0.425 –  0.103) | **-0.441**  **(-0.682 –**  **-0.021)***** | -0.133  (-0.398 –  0.133) | -0.139  (-0.404 – 0.126) | -0.086  (-0.353 – 0.180) | -0.180  (-0.444 –  0.083) | -0.022  (-0.289 – 0.246) | -0.152  (-0.417 –  0.112) | -0.131  (-0.396 – 0.135) | 0.078  (-0.189 – 0.345) |

ALS, amyotrophic lateral sclerosis; CI, confidence interval; BMI, body mass index; ALSFRS-R, amyotrophic lateral sclerosis functional rating scale-revised. * *p* < 0.05, ** *p* < 0.01, *** *p* < 0.001.

**Supplementary Table 5** Multivariate linear regression models evaluating the factors influencing the motor and cognitive phenotypes in female patients with ALS

| **Independent**  **variables**  **β(95%CI)** | **ALSFRS-R**  **score** | **Progression**  **rate** | **Total score** | **ALS-specific score** | **ALS-nonspeci-fic score** | **Language score** | **Fluency score** | **Executive score** | **Memory score** | **Visuosp-atial score** |
| --- | --- | --- | --- | --- | --- | --- | --- | --- | --- | --- |
| **rs671 (A) allele** | -0.104  (-0.369 – 0.162) | 0.101  (-0.137 – 0.340) | -0.086  (-0.314 – 0.142) | -0.044  (-0.278 – 0.191) | -0.164  (-0.396 – 0.068) | -0.102  (-0.328 – 0.124) | -0.088  (-0.342 – 0.167) | 0.033  (-0.205 – 0.272) | -0.153  (-0.388 – 0.082) | -0.103  (-0.357 – 0.152) |
| **hs-CRP** | -0.190  (-0.453 – 0.073) | 0.212  (-0.025 – 0.448) | -0.193  (-0.427 – 0.040) | -0.192  (-0.432 – 0.048) | -0.160  (-0.398 – 0.079) | -0.135  (-0.367 – 0.097) | -0.170  (-0.431 – 0.092) | -0.187  (-0.432 – 0.057) | -0.153  (-0.394 – 0.088) | -0.101  (-0.362 – 0.160) |
| **Duration** | -0.181  (-0.445 – 0.084) | **-0.422**  **(-0.660 –**  **-0.184)***** | — | — | — | — | — | — | — | — |
| **Age** | — | — | **-0.260**  **(-0.497 –**  **-0.023)*** | -0.243  (-0.487 –0.0004) | **-0.266**  **(-0.508 –**  **-0.024)*** | **-0.382**  **(-0.617 –**  **-0.147)**** | -0.036  (-0.301 – 0.229) | -0.227  (-0.475 – 0.022) | **-0.312**  **(-0.557 – -0.068)*** | 0.023  (-0.242 – 0.288) |
| **Education level** | — | — | **0.477**  **(0.238 –**  **0.716)***** | **0.448**  **(0.202 –**  **0.694)***** | **0.440**  **(0.196 –**  **0.684)***** | **0.458**  **(0.221 –**  **0.696)***** | **0.321**  **(0.053 – 0.588)*** | **0.429**  **(0.178 –**  **0.679)**** | **0.401**  **(0.155 – 0.648)**** | **0.348**  **(0.081 – 0.615)*** |

ALS, amyotrophic lateral sclerosis; ALSFRS-R, amyotrophic lateral sclerosis functional rating scale-revised; hs-CRP: high-sensitivity C-reactive protein; CI, confidence interval. * *p* < 0.05, ** *p* < 0.01, *** *p* < 0.001.

**Supplementary Table 6** The mediating role of C-reactive protein in the relationship between *ALDH2* rs671 (A) and motor and cognitive function in female patients with ALS

|  | **ALSFRS-R**  **score** | **Progression**  **rate** | **Total score** | **ALS-specific score** | **ALS-nonspeci-fic score** | **Language score** | **Fluency score** | **Executive score** | **Memory score** | **Visuosp-atial score** |
| --- | --- | --- | --- | --- | --- | --- | --- | --- | --- | --- |
| **Total effect (95% CI)** | -1.534  (-4.946 – 1.879) | 0.187  (-0.192 – 0.566) | -5.876  (-19.139 – 7.386) | -2.568  (-12.755 – 7.618) | -3.036  (-7.027 – 0.955) | -1.587  (-4.757 – 1.583) | -1.736  (-6.084 – 2.612) | 0.345  (-4.321 – 5.011) | -2.409  (-5.828 – 1.009) | -0.523  (-1.709 – 0.663) |
| **Direct effect (95% CI)** | -1.324  (-4.717 – 2.069) | 0.158  (-0.215 – 0.531) | -4.945  (-18.051 – 8.161) | -1.877  (-11.960 – 8.207) | -2.807  (-6.785 – 1.171) | -1.429  (-4.601 – 1.744) | -1.494  (-5.832 – 2.844) | 0.649  (-3.978 – 5.277) | -2.223  (-5.363 – 1.190) | -0.483  (-1.678 – 0.712) |
| **Indirect effect (95% CI)** | -0.210  (-1.226 – 0.549) | 0.029  (-0.062 – 0.195) | -0.931  (-4.265 – 2.853) | 0.692  (-3.148 – 2.292) | 0.229  (-1.378 – 0.607) | 0.158  (-0.912 – 0.558) | 0.242  (-1.038 – 1.226) | -0.304  (-1.539 – 0.757) | -0.186  (-0.983 – 0.583) | -0.040  (-0.550 – 0.116) |
| **Percent mediated (%)** | — | — | — | — | — | — | — | — | — | — |

ALDH2, aldehyde dehydrogenase 2; ALS, amyotrophic lateral sclerosis; ALSFRS-R, amyotrophic lateral sclerosis functional rating scale-revised; CI, confidence interval.

**Supplementary Table 7** Multivariate linear regression models evaluating the factors influencing the motor and cognitive phenotypes restricted to male patients in King’s clinical stage 1 to 3

| **Independent**  **variables**  **β(95%CI)** | **ALSFRS-R score** | **Progression rate** | **Total score** | **ALS-specific score** | **ALS-nonspecific score** | **Language score** | **Fluency score** | **Executive score** | **Memory score** | **Visuospatial score** |
| --- | --- | --- | --- | --- | --- | --- | --- | --- | --- | --- |
| **rs671 (A) allele** | -0.179  (-0.423 – 0.064) | **0.261**  **(0.023 – 0.499)*** | -0.159  (-0.360 – 0.042) | **-0.197**  **(-0.389 – -0.005)*** | -0.016  (-0.262 – 0.229) | -0.0002  (-0.211 – 0.210) | -0.134  (-0.386 – 0.118) | **-0.250 (-0.447 – -0.054) *** | -0.075  (-0.329 – 0.178) | -0.181  (-0.050 – 0.412) |
| **hs-CRP** | -0.166  (-0.405 – 0.072) | 0.108  (-0.126 – 0.342) | -0.195  (-0.397 – 0.007) | -0.202  (-0.394 – 0.010) | -0.131  (-0.378 – 0.115) | -0.168  (-0.379 – 0.044) | -0.093  (-0.346 – 0.160) | -0.192  (-0.389 – 0.006) | -0.080  (-0.334 – 0.175) | -0.227  (-0.459 – 0.006) |
| **alcohol use** | 0.043  (-0.189 – 0.275) | 0.004  (-0.224 – 0.231) | 0.089  (-0.105 – 0.282) | 0.103  (-0.081 – 0.287) | 0.017  (-0.219 – 0.253) | 0.120  (-0.083 – 0.322) | 0.095  (-0.147 – 0.338) | 0.056  (-0.133 – 0.245) | 0.018  (-0.225 – 0.262) | 0.005  (-0.217 – 0.227) |
| **Duration** | **-0.351**  **(-0.577 – -0.126)**** | **-0.252**  **(-0.474 – -0.031)*** | — | — | — | — | — | — | — | — |
| **Age** | — | — | **-0.458**  **(-0.642 – -0.274)***** | **-0.482**  **(-0.657 – -0.307)***** | **-0.266**  **(-0.491 – -0.042)*** | **-0.415**  **(-0.607 – -0.223)***** | -0.191  (-0.421 – 0.040) | **-0.481**  **(-0.661 – -0.301) ***** | -0.219  (-0.450 – 0.013) | **-0.279**  **(-0.490 – -0.068)*** |
| **Education level** | — | — | **0.211**  **(0.019 –**  **0.403)*** | **0.202**  **(0.019 –**  **0.385)*** | 0.178  (-0.056 –  0.412) | **0.326**  **(0.125 –**  **0.527)**** | 0.093  (-0.147 –  0.333) | 0.111  (-0.076 –  0.299) | 0.118  (-0.123 –  0.360) | **0.275**  **(0.054 –**  **0.495)*** |

ALS, amyotrophic lateral sclerosis; ALSFRS-R, amyotrophic lateral sclerosis functional rating scale-revised; hs-CRP: high-sensitivity C-reactive protein. * *p* < 0.05, ** *p* < 0.01, *** *p* < 0.001.

**Supplementary Table 8** The mediation analyses of C-reactive protein in the relationship between *ALDH2* rs671 (A) allele and motor and cognitive function restricted to male patients in King’s clinical stage 1 to 3

|  | **ALSFRS-R score** | **Progression rate** | **Total score** | **ALS-specific score** | **ALS-nonspecific score** | **Language score** | **Fluency score** | **Executive score** | **Memory score** | **Visuospatial score** |
| --- | --- | --- | --- | --- | --- | --- | --- | --- | --- | --- |
| **Total effect (95% CI)** | **-3.238**  **(-6.334 – -0.142)*** | **0.512**  **(0.132 – 0.891)**** | **-12.611**  **(-23.643 – -1.579) *** | **-11.899**  **(-20.334– -3.464)**** | -0.886  (-4.420–2.647) | -0.808  (-3.812 –2.196) | -2.631  (-6.439 –1.178) | **-8.286**  **(-13.322 – -3.249)**** | -1.335  (-4.487 –1.817) | 0.449  (-0.477 –1.374) |
| **Direct effect (95% CI)** | -2.424  (-5.715 –0.867) | **0.445**  **(0.387 –0.851)*** | -9.044  (-20.486 –2.399) | **-8.944**  **(-17.562 – -0.236)*** | -0.245  (-3.977 –3.487) | -0.002  (-3.145 –3.140) | -2.151  (-6.190 –1.888) | **-6.645**  **(-11.867 – -1.424)*** | -0.996  (-4.343 –2.350) | -0.751  (-0.208 –1.711) |
| **Indirect effect (95% CI)** | -0.814  (-2.612 –0.524) | 0.067  (-0.082 –0.260) | **-3.567**  **(-9.006 – -0.088)** | **-2.955**  **(-7.309 – -0.155)** | -0.641  (-2.506 –0.918) | **-0.806**  **(-2.208–-0.006)** | -0.480  (-1.763 –1.565) | **-1.641**  **(-4.837 – -0.065)** | -0.339  (-1.677 –0.881) | -0.303  (-0.931 –0.173) |
| **Percent mediated (%)** | — | — | 100% | 24.83% | — | — | — | 19.80% | — | — |

ALDH2, aldehyde dehydrogenase 2; ALS, amyotrophic lateral sclerosis; ALSFRS-R, amyotrophic lateral sclerosis functional rating scale-revised. * *p* < 0.05, ** *p* < 0.01.
