# Supplementary figures and images for: The role of ALDH2 rs671 polymorphism and C-reactive protein in the phenotypes of male ALS patients
Source: Front Neurosci. 2024 Sep 3;18:1397991. doi: 10.3389/fnins.2024.1397991 (PMC11405379; doi:10.3389/fnins.2024.1397991)

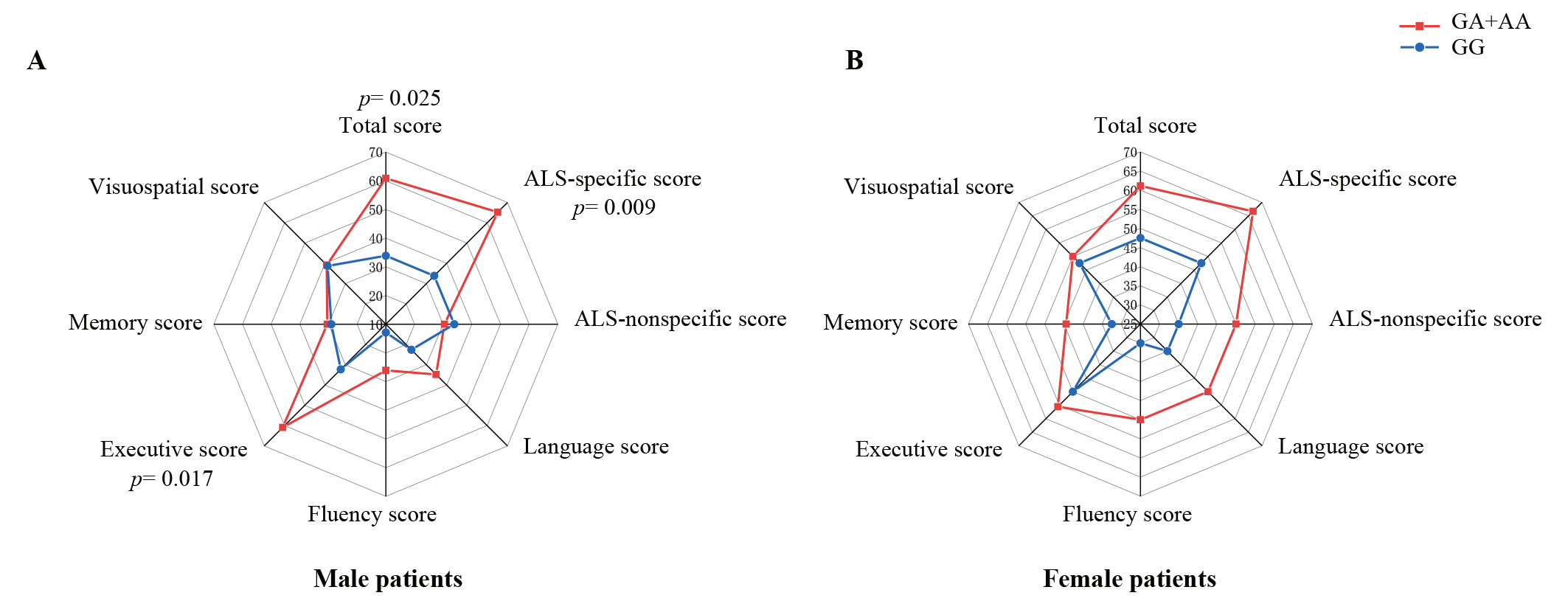

Supplement: Supplementary file 2 [file Image_1.tif]

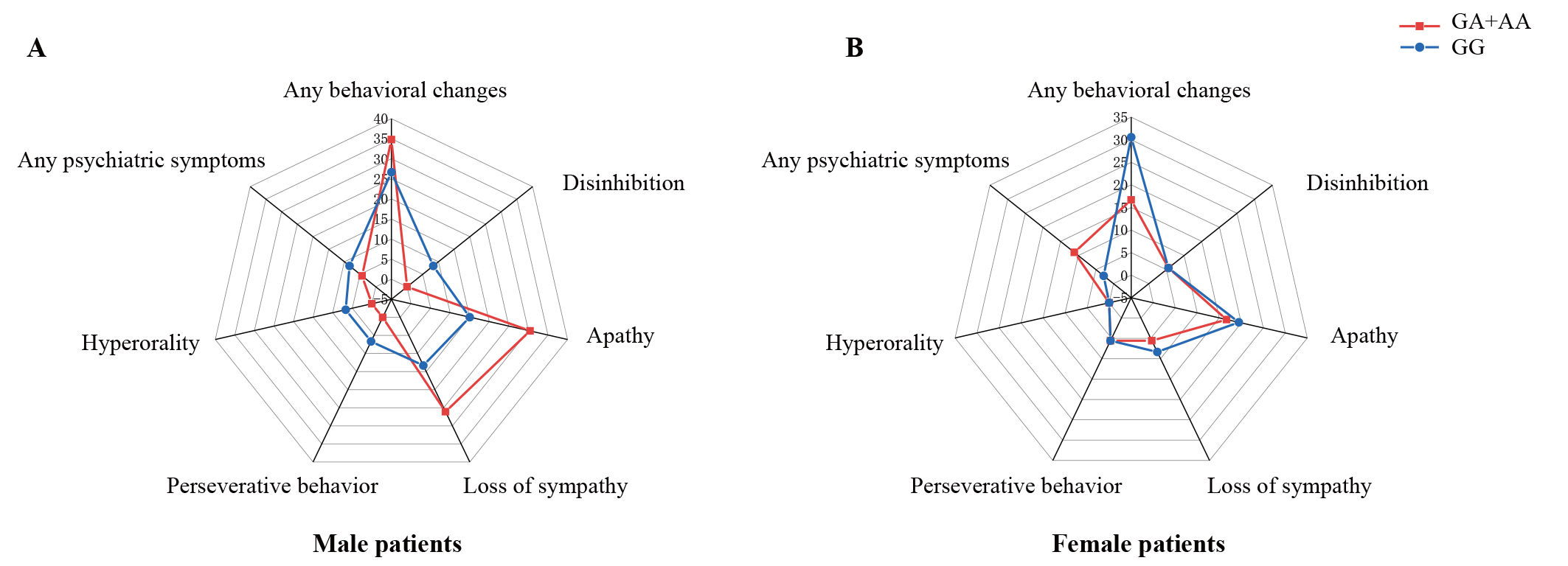

Supplement: Supplementary file 3 [file Image_2.tif]
